# Canalicular lacerations in a tertiary eye hospital: our experience with monocanalicular stents

**Published:** 2020

**Authors:** Amit Raj, Sahil Thakur, Kumar Sudesh Arya, Prem Kesarwani, Upasna Sinha

**Affiliations:** * Department of Ophthalmology, All India Institute of Medical Sciences, Patna, India; ** Department of Ocular Epidemiology, Singapore Eye Research Institute, Singapore; *** Department of Ophthalmology, Government Medical College and Hospital Chandigarh, India; **** Department of Ophthalmology, All India Institute of Medical Sciences, Delhi, India; ***** Department of Radiodiagnois, All India Institute of Medical Sciences, Patna, India

**Keywords:** canalicular injuries, monocanalicular stent, eyelid injury

## Abstract

**Introduction:** Canalicular injury is commonly encountered in lid trauma. A multitude of techniques and stents are available to manage canalicular lacerations. Monocanalicular stents offer a simple, technically easy and cost-effective solution for managing such cases.

**Objective:** This is a retrospective review of the patients presenting with canalicular lacerations to a tertiary eye hospital from January 2014 to September 2017. We evaluated factors like time of surgery, cause of injury, time of stent removal and their association with the surgical outcome. Additionally, we also reviewed the current data available in literature on the exclusive use of monocanalicular stents for the management of all types of canalicular injuries.

**Methods:** Retrospective patient file review.

**Results:** We evaluated 30 cases of canalicular injuries in 30 patients. The majority of our patients were males (24, 80%), and the mean age was 32.11±15.09 (4-59) years. The mode of injury was road traffic accidents (RTA) in 20 (66.7%), assault with sharp edged weapons in 8 (26.7%) and dog bite in 2 (6.6%) cases. The mean time of repair was 17.2±9.37 (6-36) hours after injury and the mean time of stent removal/ extrusion was 3.5±0.99 (0.5-5) months. The cases were divided based on time of repair i.e., within 24 hours (21 cases) or after 24 hours (9 cases) from the onset of injury. The extrusion rates were 14.3% (3) and 44.4% (4) respectively in the two groups. Our overall anatomical success rate was 86.7% and functional success rate of 76.7%.

**Conclusions:** Overall failure rate was 23.3% (7 out of 30). Delay in surgery (>24 hours) and dog bites were associated with a poorer prognosis of canalicular repair using monocanalicular stents.

**Abbreviations:** FDDT = Fluorescein dye disappearance test, SPSS = Statistical Package for Social Sciences, RTA = Road Traffic Accident

## Introduction

Canalicular lacerations are present in approximately 16% of all eyelid injuries [**1**]. The lacrimal canaliculi are located within the medial aspect of the eyelid and this area is different from the rest of the eyelid as it lacks a tarsal substructure. This leads to an increased propensity of avulsion due to a force that displaces the eyelid from its strong attachment at the medial canthal tendon, lacrimal, and maxillary bone [**2**]. 

Based on the mechanism of injury, canalicular lacerations are classified into direct, indirect and diffuse injuries. The direct type occurs mostly with sharp objects, indirect type due to blunt tangential forces or blows and the diffuse injury refers to extensive lid trauma associated with orbital fractures, globe rupture and other body injuries [**3**]. 

**Fig. 1:**
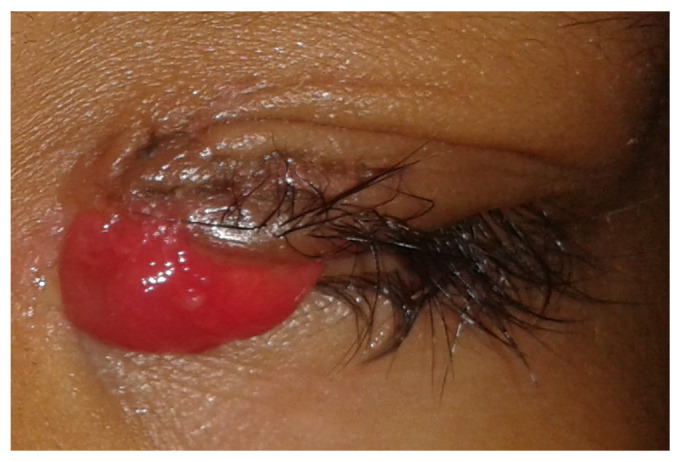
Development of granuloma and hypertrophic scar in a case without lid laceration repair

In cases in which lid trauma is inadequately managed, complications like lid margin notching, lagophthalmos, traumatic ptosis, epiphora, hypertrophic scars and wound granuloma formation can occur [**4**] (**Fig. 1**). The role of temporary intracanalicular stents in the restoration of continuity and patency of lacerated canalicular system is well documented and established [**5**,**6**]. Literature has also demonstrated that meticulous eyelid and medial canthal tendon repair, irrespective of the technique, accounts for optimal cosmetic and functional outcomes [**5**,**7**-**9**]. 

However, there is a wide range of functional success rates reported, from 61.1 to 100% [**6**,**10**-**12**]. Previously, the distance of the laceration from the punctum has been evaluated as a factor that determines post-operative success [**10**]. However, we believe that additional factors like time of surgical intervention, duration of intubation and mode of injury also influence the outcome of surgery. We undertook this study to further evaluate these factors to determine the success rate of canalicular repair using monocanalicular stents. 

## Methods

**Fig. 2:**
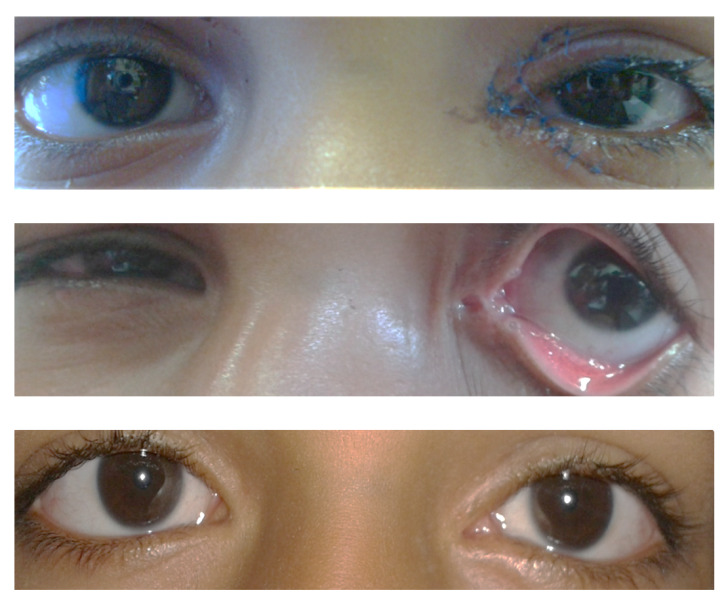
Sequential images of Bi-canalicular laceration repair at 1-day post op, 1 month and 6 months (top to bottom)

In this retrospective study, medical records of 220 patients with eyelid lacerations presenting from January 2014 to September 2017 were reviewed. Of 220 patients with eyelid lacerations, 34 (15.45%) had co-existing canalicular lacerations. We included 30 patients, 4 were excluded due to different surgical techniques/ nature of implant that was used for canalicular repair. Our study strictly adhered to the tenets of the Declaration of Helsinki and the clinical review and documentation was conducted after obtaining the Institutional Ethics Committee approval. The details of clinical history, laterality, time of presentation, type of canalicular injury, method of repair, follow up and the outcome were analyzed. All surgeries were performed by a single surgeon under local/ general anaesthesia as per need. The punctum was dilated with Nettleship’s punctum dilator. Medial lacerated end of the canaliculus was located using direct visualization under an operating microscope. In case of non-visualization of the medial cut end, different non-traumatic assisted techniques (air injection, viscoelastic and dye injection) were used to locate the lacerated end of the canaliculus. All canaliculi were repaired using monocanalicular stents (Mini Monoka, FCI Ophthalmics, Marshfield Hills, MA, USA) and peri canalicular tissue was repaired with 8-0 vicryl interrupted sutures, followed by closure of muscle, conjunctiva, and skin. Bi-canalicular lacerations were also repaired using monocanalicular stents (**Fig. 2**). Adequate instructions were given to the patients about the medications, stent care, and postoperative visits.

After the examination on the 1st postoperative day, all patients were reviewed at 1st, 4th, 8th, 12th, and 24th week. Standard post-operative regime was followed in all patients and consisted of oral/ topical antibiotics and artificial tear preparations. At each follow-up visit, the lacrimal punctum, position of the stent and ocular surface were evaluated. The stents were removed after 12-14 weeks during slit-lamp office examination, after the assessment of the patient condition (epiphora, inflammation, patient compliance and complications). In cases of stent extrusion, the time period was also noted based on patient disclosure of the episode.

After stent removal, all the patients underwent a standard fluorescein dye disappearance test (FDDT) to access the functional patency of the repaired lacrimal system. After FDDT, a gentle lacrimal irrigation with 2ml saline was performed to check the anatomical patency using a 27-gauge straight lacrimal irrigation cannula. The irrigation findings were categorized as patent (no fluid regurgitation), stenosis (patency confirmed by the patient but partial fluid regurgitation) and blocked (complete fluid regurgitation). No attempt was made to probe the canaliculus to avoid any iatrogenic injury. Complete success was defined as patent lacrimal irrigation with negative FDDT, partial success as patent irrigation or stenosis with positive FDDT and failure as blocked irrigation with positive FDDT. Both lacrimal irrigation and FDDT were repeated after 1 month of stent removal. Minimum post stent removal follow-up of 24 weeks (6 months) was ensured. Statistical analysis was performed using SPSS software (version 24, IBM, New York, USA).

## Results

Thirty eyes of 30 patients with a mean age of 32.11±15.09 (4-59 years) with canalicular injuries were included of which 80% (24) were men. The most common mode of injury was road traffic accidents (RTA) in 20 (66.7%), followed by assault with sharp edged weapons in 8 (26.7%) and dog bite in 2 (6.6%) cases. The upper canaliculus was involved in 6 (20%), lower in 20 (66.7%) and both canaliculi in 4 (13.3%). Associated medial wall fracture along with multiple orbital injuries was present in 5 (16.7%) cases. The mean time of repair was 17.2±9.37 (6-36) hours after injury. The mean time of stent removal/ extrusion was 3.5±0.99 (0.5-5) months.

The cases were divided based on time of repair i.e., within 24 hours or after 24 hours from the onset of injury. **Table 1** shows the rate of surgical success in the 2 groups. **Table 2** shows the details of the cases with tube extrusion. Most of our cases underwent early surgery and had good post-operative outcomes. 95.2% had anatomical patency on syringing and 85.7% had a physiological function with negative FDDT. The patients who were operated early also had a lower extrusion rate as compared to those who were operated late. Dog bites and delayed surgery appeared to be associated with a higher extrusion rate. 

**Table 1:** Surgical success and time of surgery (n=30)

Time of Surgery	Number of cases	Number of Tube Extrusions	Anatomical Success (Syringing)	Functional Success (FDDT)
*Within 24 hours*	21	3 (14.3%)	20 (95.2%)	18 (85.7%)
*After 24 hours*	9	4 (44.4%)	6 (66.7%)	5 (55.6%)
*Combined*	30	7 (23.3%)	26 (86.7%)	23 (76.7%)

**Table 2:** Tube Extrusion: Causes and Surgical Success (n=7)

Time of Surgery	Tube Extrusions	Nature of Injury	Time of Extrusion (weeks)	Cause	Anatomical Success (Syringing)	Functional Success (FDDT)
*Within 24 hours*	3	RTA	4	Punctal Splitting	Yes	No
		RTA	8	Spontaneous Extrusion	Yes	Yes
		Assault	8	Spontaneous Extrusion	No	Yes
*After 24 hours*	4	Dog Bite	4	Spontaneous Extrusion	Yes	No
		Dog Bite	6	Spontaneous Extrusion	No	No
		RTA	8	Wound Granuloma	No	No
		Assault	1	Spontaneous Extrusion	No	No

## Discussion

In our study, most of the cases were young males, this being similar to the previous studies [**11**,**13**,**14**]. The lower canaliculi were involved in 20 (66.7%) cases, this trend being also similar to reports published earlier [**11**,**14**] (**Table 3**). 

**Table 3:** Summary of studies exclusively using monocanalicular stents for canalicular laceration repair

Author	Study Population (Number, Gender, Age)	Canalicular Involvement	Mechanism of Injury	Type of Stent	Extrusion Rate	Anatomical Success	Complications
*Naik et al. [**11**]*	24, 20 males (83.3%) 16 years (10 months-52 years)	Upper: 8 Lower: 13 Bi-Canalicular: 3	Blouse hook fastener: 20.8% Metal Rod: 20.8% Bicycle Handle: 16.7%	Mini Monoka	3 (11.1%)	Anatomical Success: 18 (90%) Functional Success: 20 (100%)	Spontaneous Extrusion: 3
*Lee et al. [**13**]*	36, 26 males (72%), 34 years (1-64 years)	Upper: 10 Lower: 26		Mini Monoka	2 (6%)	Anatomical Success: 16 (94.12%) Functional Success: 14 (82.35%)	Spontaneous Extrusion: 2, Punctal Slits: 2
*Eo et al. [**21**]*	15 patients, 17 eyes			Monostent and Mini Monoka	1 (5.88%)	Anatomical Success: 16 (94.12%) Functional Success: 14 (82.35%)	Stent Extrusion: 1
*Chowdhury et al. [**14**]*	61, 46 males (75%), 27 years (1-89 years)	Upper: 11 Lower: 46 Bi-Canalicular: 4	Punch 28%, Falls 12%, Broken Glass 10%	Mini Monoka	9 (15%)	Functional Success: 56 (92%)	Stent Extrusion: 9 Symptomatic Failure: 5
*This study*	30, 24 males (80%), 32 years (4-59 years)	Upper: 6 Lower: 20 Bi-Canalicular: 4	RTA: 20, Assault: 8, Dog Bite: 2	Mini Monoka	7 (23.3%)	Anatomical Success: 26 (86.7%) Functional Success: 23 (76.7%)	Spontaneous Extrusion: 5, Punctal Splitting: 1, Granuloma: 1

The primary mode of injury was trauma due to road side accidents in our study, this being similar to the study of Singh et al. who demonstrated a comparable demographic and surgical success profile like the one in our study [**10**]. In a study of 36 patients, they reported RTA in 47.22% of the patients. Monocanalicular stents were used in 91.67% of their cases and they reported an anatomical and functional success rate of 77.78% and 61.11% respectively. 

**Fig. 3:**
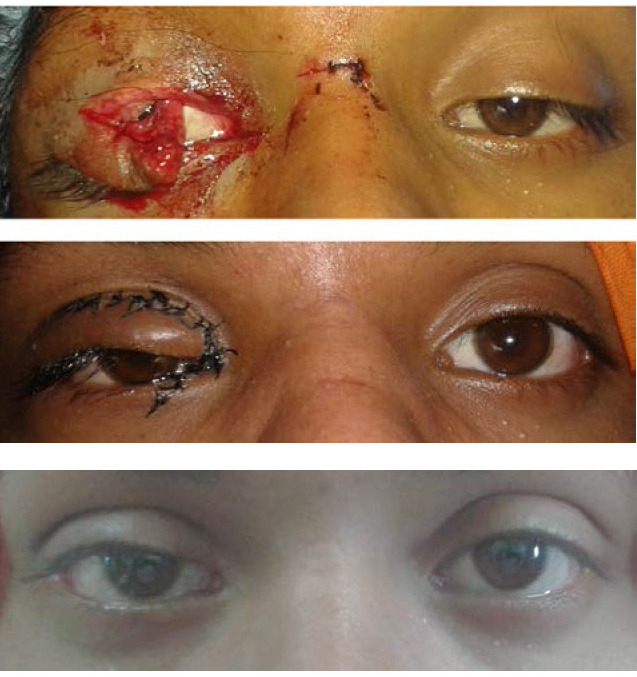
Sequential images of Monocanalicular laceration repair at presentation, 1 week and 3 months (top to bottom)

For the identification of the lacerated medial end of the canaliculi, we used magnification on the operating microscope to locate the other end. In case of non-visualization, non-traumatic assisted techniques like air injection, viscoelastic and dye injection were employed from the other end to identify the proximal end of the laceration [**12**]. These techniques are usually required only in cases in which repair is delayed beyond 24 hours, due to tissue retraction and canalicular collapse. We have used the technique previously described by Naik et al. in the repair of bi-canalicular lacerations [**15**]. In our experience, the monocanalicular stents are effective, less invasive, patient friendly and technically easy to remove than conventional bi-canalicular stents (**Fig. 3**).

Previously, authors have described associated ocular injuries in patients with canalicular lacerations like subconjunctival haemorrhage, traumatic hyphaema, retinal oedema, orbital wall fracture, traumatic optic neuropathy and traumatic cataracts [**13**]. In our study, 5 cases (16.7%) had medial orbital wall fracture and 1 (3.3%) had simultaneous globe perforation that was surgically repaired [**11**]. There were 2 cases with dog bite related injuries and it was a significant factor for surgical failure (P<0.001). It has been shown that in cases of periocular dog bite wounds, 66% involve the canaliculi [**16**]. In such cases, it is important to prevent other serious and potentially life-threatening complications like sepsis, arthritis, osteomyelitis, compartment syndrome, loss of a limb and rabies [**17**]. In our study, we had anatomical success in one case, while functional success could not be achieved in any of the cases. Both cases were children (4 and 6 years old), had associated mid facial lacerations, were operated after 24 hours from the injury and later developed spontaneous tube extrusion at around 2 weeks from surgery. This occurred despite broad spectrum systemic antibiotics and regular follow up. Wound infection in dog bites is well known and still remains an important cause of surgical failure [**16**]. Early detection of specific canine oral pathogens and institution of targeted treatment may however give better results [**18**,**19**]. 

Murchison et al. have previously shown that the success rate for canalicular laceration repair is dependent on surgical skill [**20**]. In our study, a single experienced surgeon performed all the surgeries under local/ general anaesthesia. The distal location of the canalicular laceration from the punctum has also been shown to have a better surgical success rate, however, we did not assess this parameter in our study [**10**]. 

Our study has an anatomical success rate of 86.7% and a functional success rate of 76.7%, which is similar to the studies published earlier [**9**,**12**-**14**]. Bivariate analysis of the data using Spearman correlations indicated that the anatomical success (S=0.381, P<0.05) and functional success (S=0.711, P<0.01) was correlated with surgery when performed before 24 hours. The age, gender, laterality, canalicular location, time of tube removal and presence of associated injuries were not statistical predictors of surgical success (P>0.05). Our results indicated that the time of surgical intervention is an important predictor of final surgical success. Chowdhury et al. have previously reported that 77% of the repairs were conducted within 24 hours from injury; 15% were within 2 days and 8% later on. Subsequently, on analyzing their failure cases (5, 8%), all except one case were repaired on the first day. Of the 5 patients 3 suffered glass injuries, 1 a fall, 1 was kicked and canalicular repair was performed by a fellow in 4 of 5 cases [**14**]. However, in our study, all the patients were operated by an experienced surgeon, yet underwent surgical failure, indicating a role of mechanism of injury/ timing of surgery in determining the final surgical outcome. Nonetheless, further studies are needed to assess this association. 

Yet, there are certain limitations of our study including the lack of a control group, small and unequal sample size and a retrospective nature. But, the epidemiological profile of the canalicular lacerations makes it difficult to design a prospective study. 

## Conclusion

This study is a novel attempt to assess the outcome predictors of canalicular laceration repair using monocanalicular stents in a tertiary eye hospital. These stents can also be used successfully in cases with bi-canalicular lacerations. However, it is imperative to take up such cases at the earliest to optimize easy canalicular visualization without much mechanical intervention like the use of probes or need of bi-canalicular stents. However, dog bites are still a challenge for surgeons and need patient optimization with adequate antibiotic coverage before intervention.

**Conflict of Interest**

All authors declare that they have no conflict of interest.

**Ethical approval**

All procedures performed in studies involving human participants were in accordance with the ethical standards of the institutional and/ or national research committee and with the 1964 Helsinki Declaration and its later amendments or comparable ethical standards.

**Informed consent**

An informed consent was obtained from all individual participants included in the study.

**Sources of funding**

None.

**Financial disclosure**

None.
